# Safety signals and CMV-related events reported during letermovir therapy: a FAERS database study

**DOI:** 10.3389/fphar.2026.1935778

**Published:** 2026-09-29

**Authors:** Zhangjie Chen, Shuning Zhang, Sisi Zhen, Chun Wang, Xin Chen, Sizhou Feng

**Affiliations:** 1 Tianjin Institutes of Health Science, State Key Laboratory of Experimental Hematology, National Clinical Research Center for Blood Diseases, Institute of Hematology and Blood Diseases Hospital, Chinese Academy of Medical Sciences and Peking Union Medical College, Tianjin, China; 2 College of Clinical Medicine for Obstetrics and Gynecology and Pediatrics, Fujian Medical University, Fuzhou, China; 3 Department of Hematology, The Affiliated Cancer Hospital of Zhengzhou University and Henan Cancer Hospital, Zhengzhou, China

**Keywords:** adverse drug effect, CMV infection, FAERS, HSCT, letermovir

## Abstract

**Background:**

Letermovir is an antiviral agent approved for CMV infection prophylaxis in transplant recipients. This study aimed to evaluate safety signals reported during letermovir treatment, using data from the FDA Adverse Event Reporting System (FAERS).

**Methods:**

Data from October 2017 to September 2024 were extracted from FAERS database. Reported events were analyzed across hierarchy levels, from Preferred Term (PT) to High-Level Terms (HLTs) and System Organ Classes (SOCs). Disproportionality analysis was performed to detect safety signals. Recorded time to onset was analyzed descriptively, and univariable logistic regression was performed to explore report-level factors.

**Results:**

The most frequently reported events was infections and infestations at SOC level, cytomegalovirus infection at HLT level and CMV infection reactivation at Preferred Term level. Apart from CMV-related events, safety signals involved hematological abnormalities, pericardial effusion, hepatic and renal failure, bacterial and viral infections, pathogen resistance, and transplantation-related events. The median recorded time to onset was 25 days for all events, and 33 days for CMV-related events, with later recorded onset in intravenous than tablet reports. Exploratory logistic regression showed lower odds of CMV-related event reporting with older age and treatment initiation in more recent years, higher odds with intravenous administration, and regional differences in reporting.

**Conclusion:**

This study identified safety signals reported with letermovir involving hematological, cardiac, hepatic, renal, infectious, and transplantation-related events. CMV-related signals may reflect breakthrough infection. These findings require validation in clinical studies.

## Introduction

Letermovir is an antiviral agent that targets human cytomegalovirus (CMV) by inhibiting the CMV DNA terminase complex (Melendez and Razonable, 2015), a mechanism distinct from other CMV therapies. It is approved for the prevention of CMV infection and disease in CMV-seropositive recipients of allogeneic hematopoietic stem cell transplantation (HSCT) and has become a standard of care in this population, where CMV reactivation significantly increases morbidity and mortality (Shigle et al., 2020; Lin et al., 2021; Piñana et al., 2024). In addition to HSCT, letermovir is approved for the prevention of CMV diseases in high-risk donor-seropositive/recipient-seronegative kidney transplantation recipients, and has been increasingly used in solid organ transplant (SOT), due to its favorable safety profile and efficacy in reducing CMV-related complications (El Helou and Razonable, 2019; Winstead et al., 2021; Limaye et al., 2023). Previous studies have also suggested that letermovir may have additional benefits, such as modulating immune reconstitution and potentially reducing the risk of graft-versus-host disease (GVHD) (Moore et al., 2023; Zamora et al., 2021a; Lorentino et al., 2022; Spadea et al., 2026).

Letermovir has demonstrated strong clinical efficacy and a favorable safety profile (Marty et al., 2017; Russo et al., 2024), however, its adverse drug reactions (ADRs) still require careful consideration by physicians and patients. According to prescribing information, common side effects include nausea, diarrhea, vomiting, peripheral edema, cough, headache, fatigue and abdominal pain (Merck and Co, 2025a). Additionally, cardiac adverse events, including tachycardia and atrial fibrillation, were observed at a higher incidence compared to placebo in adult HSCT recipients (Merck and Co, 2025b). Previous clinical trials and retrospective studies have reported other serious adverse events during letermovir treatment, including recurrent leukemia, pneumonia, pruritus, cytopenia and increased incidence of EBV reactivation (Russo et al., 2024; Robin et al., 2020; Kong et al., 2024), although most were categorized as unrelated to letermovir. Furthermore, CMV breakthrough infections and the development of CMV resistance have been reported during letermovir prophylaxis (Robin et al., 2020; Alain et al., 2020; Khawaja et al., 2023), indicating the need for further investigation into the risk factors and clinical implications.

Given the expanding use of letermovir in and beyond HSCT patients, understanding its real-world safety profile is critical. This study used data from the United States Food and Drug Administration Adverse Event Reporting System (FAERS) to identify safety signals reported with letermovir and to characterize the recorded time to onset and report-level factors associated with CMV-related events during treatment.

## Methods

### Data source

All data was sourced from FAERS (https://www.fda.gov/drugs/surveillance/fdas-adverse-event-reporting-system-faers), a publicly accessible database for post-marketing surveillance of drug and therapeutic biologic products. ADRs in this database are recorded using Preferred Term (PT) from the Medical Dictionary for Regulatory Activities (MedDRA). MedDRA (https://www.meddra.org/how-to-use/support-documentation/english/welcome) is a globally standardized medical terminology structured into five hierarchical levels, ranging from Lowest Level Term (LLT) to PT, High-Level Terms (HLTs), High-Level Group Terms (HLGTs), and System Organ Classes (SOCs). In this study, we used PT along with corresponding HLT and SOC to describe and analyze ADRs.

### Data processing

FAERS data, provided quarterly, were downloaded in ASCII format for the period from October 2017 to September 2024, as letermovir received FDA approval on 9 November 2017. All ADR reports listing drugs as “PS” (primary suspect drug) were selected, reports related to letermovir were identified by filtering drug names containing “LETERMOVIR” or “PREVYMIS.” Since letermovir is primarily used in HSCT recipients, ADRs reports of drugs used in bone marrow transplantation (BMT) were extracted for comparative analysis by filtering drug indication for “Bone marrow transplant” and “Stem cell transplant”. Duplicate reports were removed following FDA’s recommendations: when CASEIDs were identical, the record with the most recent FDA_DT was retained; if both the CASE ID and FDA_DT were the same, the record with the highest PRIMARYID was selected. To ensure data relevance, unrelated reports, including product issue and dose changes were excluded. Finally, the PTs were mapped to their corresponding HLTs and primary SOCs according to MedDRA version 26.1 (Figure 1).

**FIGURE 1:**
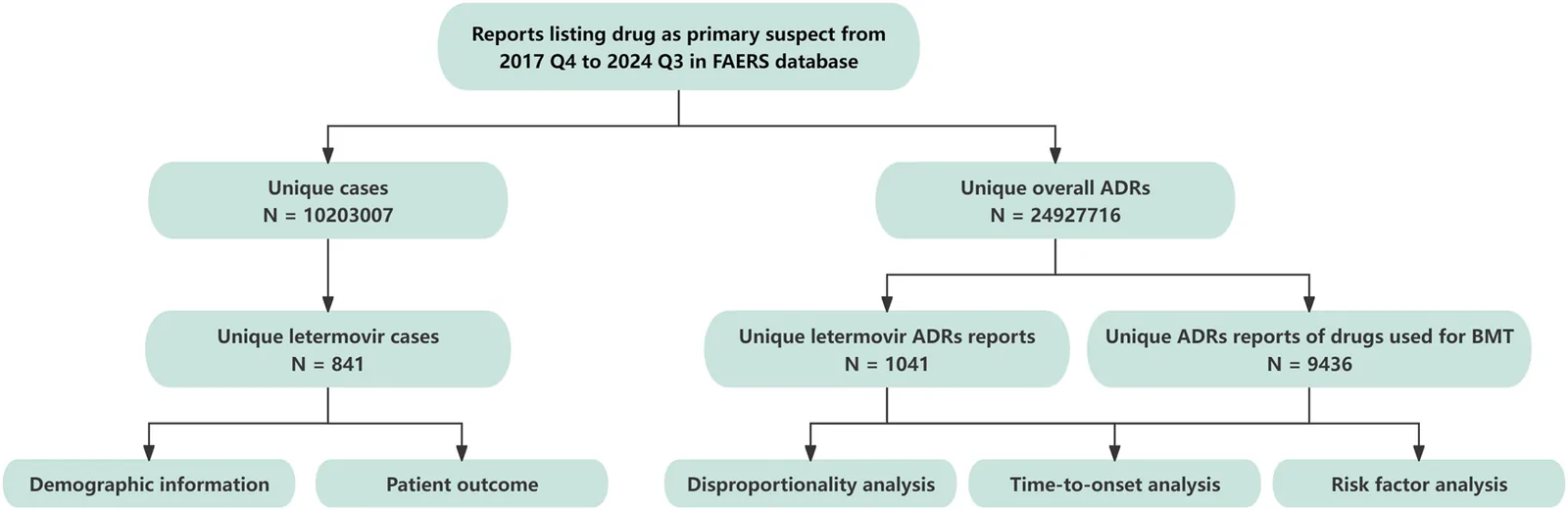
Flowchart of data processing and analysis.

### Statistical analysis

All statistical analyses were performed using R language programming (version 4.4.1, R Development Core Team). Reports of letermovir also included CMV infection events occurred during treatment, these reports were regarded as CMV recurrence or breakthrough infections instead of drug toxicity or adverse effects. Therefore, events associated with CMV infections were selected for further analysis (Supplementary Table S1). Recorded time-to-onset was analyzed descriptively using Kaplan-Meier curves, and log-rank test was used for comparison. Univariable logistic regression model was used to explore report-level factors associated with death and life-threatening outcomes and with the reporting of CMV-related events during treatment. Results were presented as odds ratio (OR) with 95% confidence intervals (95% CI).

Disproportionality analysis was performed using Reporting Odds Ratio (ROR), Proportional Reporting Ratio (PRR), Empirical Bayes Geometric Mean (EBGM), and Bayesian Confidence Propagation Neural Network (BCPNN) to identify significant signals, and the formulas were detailed in Table 1. These methods compare the observed and expected frequencies of events to detect potential safety signals of the drug. Significant signals were identified using the following thresholds: ROR >2 with lower 95% confidence interval (CI) > 1, PRR >2 with lower 95% CI > 1, EB05 > 2, and IC025 > 0. To ensure accuracy, events meeting all criteria were considered significant signals. All tests were two-sided, and a P < 0.05 was considered statistically significant. In this pharmacovigilance study, a significant signal indicated a statistical drug-events reporting association that warrants further investigation. Importantly, significant signals do not establish causality, but rather guides where more in-depth investigation is necessary.

**TABLE 1:** Calculation formula and significance criteria for ADRs.

Method	Calculation Formula	Significance Criteria
ROR	*ROR* = (*a/c*) / (*b/d*) 95% *CI* = exp(ln(*ROR*) ± 1.96√(1/*a* +1/*b* + 1/*c* + 1/*d*))	ROR > 2 and lower 95% CI > 1
PRR	*PRR* = (*a*/(*a* + *c*))/(*b*/(*b* + *d*)) 95% *CI* = exp(ln(*PRR*) ± 1.96√(1/*a*-1/(*a* + *c*) + 1/*b*-1/(*b* + *d*)))	RPR > 2 and lower 95% CI > 1
EBGM	$\mathrm{EBGM}=\mathrm{aN}/\left(\left(a+b\right)\left(a+c\right)\right)$ $95\%\;\mathrm{CI}=\exp\left(\ln\left(\mathrm{EBGM}\right)\pm 1.96\surd \left(1/a+1/b+1/c+1/d\right)\right)$	EBGM05 > 2
BCPNN	${IC=\log}_{2}\left(aN/\left(\left(a+b\right)\left(a+c\right)\right)\right)$ ${\gamma =\gamma }_{11}(\left(N+\alpha \right)\left(N+\beta \right))/\left(\left(a+c+\alpha_{1}\right)\left(a+b+\beta_{1}\right)\right)$ $\left.E\left(IC\right)=\log_{2}(\left(\left(a+\gamma_{11}\right)\left(N+\alpha \right)\left(N+\beta \right)\right)/\left(\left(N+\gamma \right)\left(a+c+\alpha_{1}\right)\left(a+b+\beta_{1}\right)\right)\right)$ $V\left(IC\right)=1/{\left(\ln\left(2\right)\right)}^{2}\left(\left(N-a+\gamma -\gamma_{11}\right)/\left(\left(a+\gamma_{11}\right)\left(1+N+\gamma \right)\right)+\left(N-\left(a+c\right)+\alpha -\alpha_{1}\right)/\left(\left(a+c+\alpha_{1}\right)\left(1+N+\alpha \right)\right)+\left(N-\left(a+b\right)+\beta -\beta_{1}\right)/\left(\left(a+b+\beta_{1}\right)\left(1+N+\beta \right)\right)\right)$ ${IC}_{025}=E\left(IC\right)-2\surd \left(V\left(IC\right)\right)$	IC025 > 0

ADR: adverse drug reaction; ROR: Reporting Odds Ratio; PRR: Proportional Reporting Ratio; EBGM: Empirical Bayes Geometric Meanl IC: Information Component. a = ADR for the target drug, b = ADR for other drugs, c = Non-ADR for the target drug, d = Non-ADR for other drugs.

## Results

### Baseline clinical characteristics

During the study period, a total of 11,990,882 cases were reported, 10,203,007 cases were included after eliminating duplication. Of these reports, 841 cases involved letermovir treatment (Table 2). The median age was 53 (IQR: 35.5–62.0) years old, with 320 females (38.0%), 403 males (47.9%) and 118 cases with unknown gender (14.0%). The top three reporting countries were United States (n = 456, 54.2%), Japan (n = 129, 15.3%) and France (n = 55, 6.5%). The most common sources of reports were customers (n = 231, 27.5%) and physicians (n = 230, 27.3%). The highest number of cases was recorded in 2019 (155, 18.4%). In terms of patient outcome, severe events included 167 hospitalization (19.9%), 160 deaths (19.0%), 9 life-threatening (1.1%), and five disability events (0.6%). Additionally, other serious events were recorded in 224 cases (26.6%), and 276 cases (32.8%) lacked outcome data. The peak in death cases occurred in 2021 (30/160, 18.8%). Logistic regression showed that among letermovir reports, older age was associated with higher odds of reported death and life-threatening outcomes (OR = 1.015, 95% CI = 1.004–1.027), while higher body weight (OR = 0.982, 95% CI = 0.964–0.999) and more recent reporting years (OR = 0.839, 95% CI = 0.741–0.947) were associated with lower odds. Compared with other regions, reports from North America had lower odds of these outcomes (OR = 0.350, 95% CI = 0.240–0.503), whereas cases from Asia had higher odds (OR = 2.939, 95% CI = 2.011–4.280) (Figure 2).

**TABLE 2:** Clinical characteristics of cases with letermovir as the primary suspect drug.

Item	Number (n = 841)	Proportion, %
Age (years)		
Median (IQR)	53 (35.5-62)	
Mean (SD)	47.7 (18.9)	
<18	55	6.5%
18-44	149	17.7%
45-64	253	30.1%
>64	110	13.1%
NA	274	32.6%
Gender		
Male	403	47.9%
Female	320	38.0%
Unknown	118	14.0%
Weight (kg)		
Median (IQR)	58.8 (50.0-73.9)	
Mean (SD)	61.8 (23.7)	
Report year		
2018	49	5.8%
2019	155	18.4%
2020	122	14.5%
2021	128	15.2%
2022	125	14.9%
2023	142	16.9%
2024	120	14.3%
Reporter’s occupation (code)		
Customer (CN)	231	27.5%
Health professional (HP)	158	18.8%
Pharmacist (PH)	128	15.2%
Physician (MD)	230	27.3%
Other health professional (OT)	38	4.5%
NA	56	6.7%
Occurring country/territory (code)		
Argentina (AR)	5	0.6%
Austria (AT)	2	0.2%
Australia (AU)	4	0.5%
Belgium (BE)	4	0.5%
Brazil (BR)	10	1.2%
Canada (CA)	6	0.7%
Switzerland (CH)	6	0.7%
China (CN)	7	0.8%
Czechia (CZ)	3	0.4%
Germany (DE)	17	2.0%
Spain (ES)	27	3.2%
France (FR)	51	6.0%
United Kingdom (GB)	8	1.0%
Greece (GR)	1	0.1%
Croatia (HR)	16	1.9%
Italy (IT)	12	1.4%
Japan (JP)	129	15.3%
South Korea (KR)	30	3.6%
Netherlands (NL)	5	0.6%
Norway (NO)	1	0.1%
Poland (PL)	5	0.6%
Portugal (PT)	2	0.2%
Serbia (RS)	6	0.7%
Slovenia (SI)	4	0.5%
Taiwan (TW)	7	0.8%
United States (US)	393	46.7%
NA	80	9.5%
Outcome (code)		
Death (DE)	160	19.0%
Life-Threatening (LT)	9	1.1%
Hospitalization-Initial or Prolonged (HO)	167	19.9%
Disability (DS)	5	0.6%
Other Serious (Important Medical Event)	224	26.6%
NA	276	32.8%

IQR: interquartile range; SD: standard deviation; NA: not available.

**FIGURE 2:**
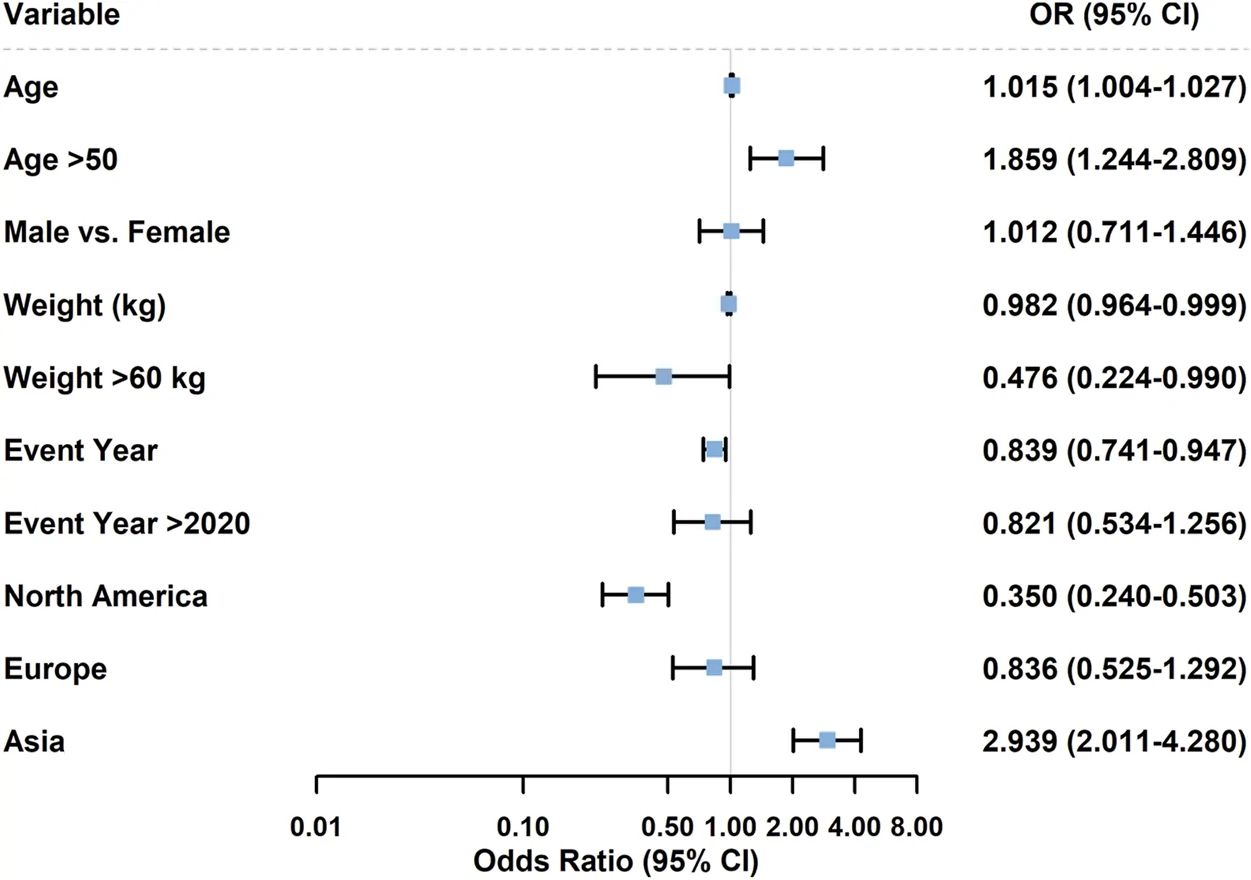
Exploratory univariable logistic regression of report-level factors associated with death and life-threatening outcomes among letermovir reports.

### Adverse drug reaction characteristics

After excluding unrelated adverse reaction reports, 1041 adverse reactions were analyzed, with a median of 1 (range: 1–22) ADR per case. ADRs reported with letermovir with ≥3 PT reports were presented in Table 3, along with the corresponding HLT and SOC. The five most frequent SOCs were infections and infestations (317, 30.5%), gastrointestinal disorders (98, 9.4%), immune system disorder (91, 8.7%), investigations (77, 7.4%) and general disorders and administration site conditions (76, 7.3%). At the HLT level, CMV infection was most frequent (158, 15.2%), followed by immune and associated condition not elsewhere classified (NEC) (82, 7.9%), infections NEC (41, 3.9%), renal failure and impairment (35, 3.4%) and general signs and symptoms NEC (34, 3.2%). Within the CMV-related events at HLT level, the most frequently reported PTs were CMV infection reactivation (62, 6.0%), CMV infection (57, 5.5%), CMV viraemia (12, 1.2%), CMV test positive (11, 1.1%) and CMV enterocolitis (8, 0.8%). Co-occurring HLTs, notably CMV infections with immune-associated conditions NEC (2.4%, n = 25), were displayed in Figure 3A and Supplementary Table S2. At PT level, CMV infection reactivation (62, 6.0%) and CMV infection (57, 5.5%) remained the most frequently reported ADRs, alongside pathogen resistance (31, 3.0%), GVHD (21, 2.0%), and acute GVHD in skin (16, 1.5%). The most frequent co-occurring PTs included pathogen resistance with CMV infection (4, 0.4%), acute GVHD with CMV infection (4, 0.4%), viral load increased with pathogen resistance (4, 0.4%), and vomiting with nausea (4, 0.4%) (Figure 3B; Supplementary Table S3).

**TABLE 3:** Number and percentage of letermovir ADRs with ≥3 reports at PT level.

SOC			HLT			PT		
Terminology	Count	Percentage	Terminology	Count	Percentage	Terminology	Count	Percentage
Blood and lymphatic system disorders	44	4.23%	Coagulopathies	6	0.58%	Thrombotic microangiopathy	6	0.58%
			Marrow depression and hypoplastic anaemias	14	1.34%	Pancytopenia	7	0.67%
			Neutropenias	12	1.15%	Agranulocytosis	4	0.38%
						Neutropenia	6	0.58%
			Thrombocytopenias	6	0.58%	Thrombocytopenia	6	0.58%
Cardiac disorders	34	3.27%	Heart failures NEC	8	0.77%	Cardiac failure	5	0.48%
			Pericardial disorders NEC	3	0.29%	Pericardial effusion	3	0.29%
			Supraventricular arrhythmias	10	0.96%	Atrial fibrillation	8	0.77%
			Ventricular arrhythmias and cardiac arrest	5	0.48%	Cardiac arrest	4	0.38%
Gastrointestinal disorders	98	9.41%	Colitis (excl infective)	5	0.48%	Colitis	4	0.38%
			Diarrhoea (excl infective)	9	0.86%	Diarrhoea	9	0.86%
			Gastrointestinal and abdominal pains (excl oral and throat)	8	0.77%	Abdominal pain	3	0.29%
						Abdominal pain upper	5	0.48%
			Gastrointestinal signs and symptoms NEC	11	1.06%	Dysphagia	11	1.06%
			Nausea and vomiting symptoms	19	1.83%	Nausea	13	1.25%
						Vomiting	6	0.58%
			Non-site specific gastrointestinal haemorrhages	7	0.67%	Haematochezia	4	0.38%
			Stomatitis and ulceration	7	0.67%	Stomatitis	6	0.58%
General disorders and administration site conditions	76	7.30%	Asthenic conditions	11	1.06%	Fatigue	9	0.86%
			Febrile disorders	11	1.06%	Pyrexia	11	1.06%
			General signs and symptoms NEC	34	3.27%	Condition aggravated	3	0.29%
						Disease progression	4	0.38%
						Disease recurrence	4	0.38%
						General physical health deterioration	5	0.48%
						Ill-defined disorder	3	0.29%
						Multiple organ dysfunction syndrome	8	0.77%
			Mucosal findings abnormal	4	0.38%	Mucosal inflammation	3	0.29%
			Oedema NEC	12	1.15%	Oedema	5	0.48%
						Oedema peripheral	5	0.48%
Hepatobiliary disorders	14	1.34%	Hepatic failure and associated disorders	5	0.48%	Hepatic failure	5	0.48%
Immune system disorders	91	8.74%	Immune and associated conditions NEC	82	7.88%	Acute graft versus host disease	11	1.06%
						Acute graft versus host disease in intestine	7	0.67%
						Acute graft versus host disease in skin	16	1.54%
						Engraftment syndrome	5	0.48%
						Graft versus host disease	21	2.02%
						Graft versus host disease in gastrointestinal tract	7	0.67%
						Graft versus host disease in skin	5	0.48%
Infections and infestations	317	30.45%	Bacterial infections NEC	12	1.15%	Bacterial infection	3	0.29%
						Bacterial sepsis	5	0.48%
			Coronavirus infections	9	0.86%	COVID-19	8	0.77%
			Cytomegaloviral infections	158	15.18%	Cytomegalovirus colitis	5	0.48%
						Cytomegalovirus enterocolitis	8	0.77%
						Cytomegalovirus gastroenteritis	3	0.29%
						Cytomegalovirus infection	57	5.48%
						Cytomegalovirus infection reactivation	62	5.96%
						Cytomegalovirus viraemia	12	1.15%
						Pneumonia cytomegaloviral	5	0.48%
			Herpes viral infections	15	1.44%	Gastritis herpes	3	0.29%
						Human herpesvirus 6 encephalitis	3	0.29%
						Human herpesvirus 6 infection	4	0.38%
			Infections NEC	41	3.94%	Infection	8	0.77%
						Pathogen resistance	31	2.98%
			Lower respiratory tract and lung infections	9	0.86%	Pneumonia	9	0.86%
			Sepsis, bacteraemia, viraemia and fungaemia NEC	13	1.25%	Sepsis	4	0.38%
						Septic shock	7	0.67%
			Urinary tract infections	3	0.29%	Urinary tract infection	3	0.29%
Injury, poisoning and procedural complications	32	3.07%	Medication errors, product use errors and issues NEC	8	0.77%	Circumstance or information capable of leading to medication error	7	0.67%
			Poisoning and toxicity	4	0.38%	Toxicity to various agents	4	0.38%
			Transplantation complications	11	1.06%	Engraft failure	8	0.77%
Investigations	77	7.40%	Renal function analyses	7	0.67%	Blood creatinine increased	4	0.38%
			Vascular tests NEC (incl blood pressure)	7	0.67%	Blood pressure decreased	3	0.29%
						Blood pressure increased	3	0.29%
			Virus identification and serology	27	2.59%	Cytomegalovirus test positive	11	1.06%
						Viral load increased	8	0.77%
						Viral mutation identified	3	0.29%
			White blood cell analyses	8	0.77%	White blood cell count decreased	4	0.38%
Neoplasms benign, malignant and unspecified (incl cysts and polyps)	28	2.69%	Leukaemias acute lymphocytic	3	0.29%	Acute lymphocytic leukaemia recurrent	3	0.29%
			Leukaemias acute myeloid	12	1.15%	Acute myeloid leukaemia	5	0.48%
						Acute myeloid leukaemia recurrent	7	0.67%
Nervous system disorders	30	2.88%	Headaches NEC	3	0.29%	Headache	3	0.29%
			Peripheral neuropathies NEC	3	0.29%	Neuropathy peripheral	3	0.29%
Renal and urinary disorders	40	3.84%	Bladder infections and inflammations	3	0.29%	Cystitis haemorrhagic	3	0.29%
			Renal failure and impairment	35	3.36%	Acute kidney injury	10	0.96%
						Renal failure	13	1.25%
						Renal impairment	12	1.15%
Respiratory, thoracic and mediastinal disorders	27	2.59%	Breathing abnormalities	5	0.48%	Respiratory distress	3	0.29%
			Respiratory failures (excl neonatal)	5	0.48%	Respiratory failure	5	0.48%
Skin and subcutaneous tissue disorders	16	1.54%	Rashes, eruptions and exanthems NEC	7	0.67%	Rash	7	0.67%
Social circumstances	6	0.58%	Economic circumstances	5	0.48%	Inability to afford medication	5	0.48%
Surgical and medical procedures	59	5.67%	Gastrointestinal therapeutic procedures NEC	6	0.58%	Gastrointestinal tube insertion	6	0.58%
			Haematological therapeutic procedures NEC	24	2.31%	Bone marrow transplant	15	1.44%
						Stem cell transplant	9	0.86%
			Therapeutic procedures NEC	18	1.73%	Therapy interrupted	8	0.77%
						Transplant	6	0.58%

SOC: System organ classes; HLT: High-level terms; PT: Preferred terms; NES: Not-elsewhere classified.

**FIGURE 3:**
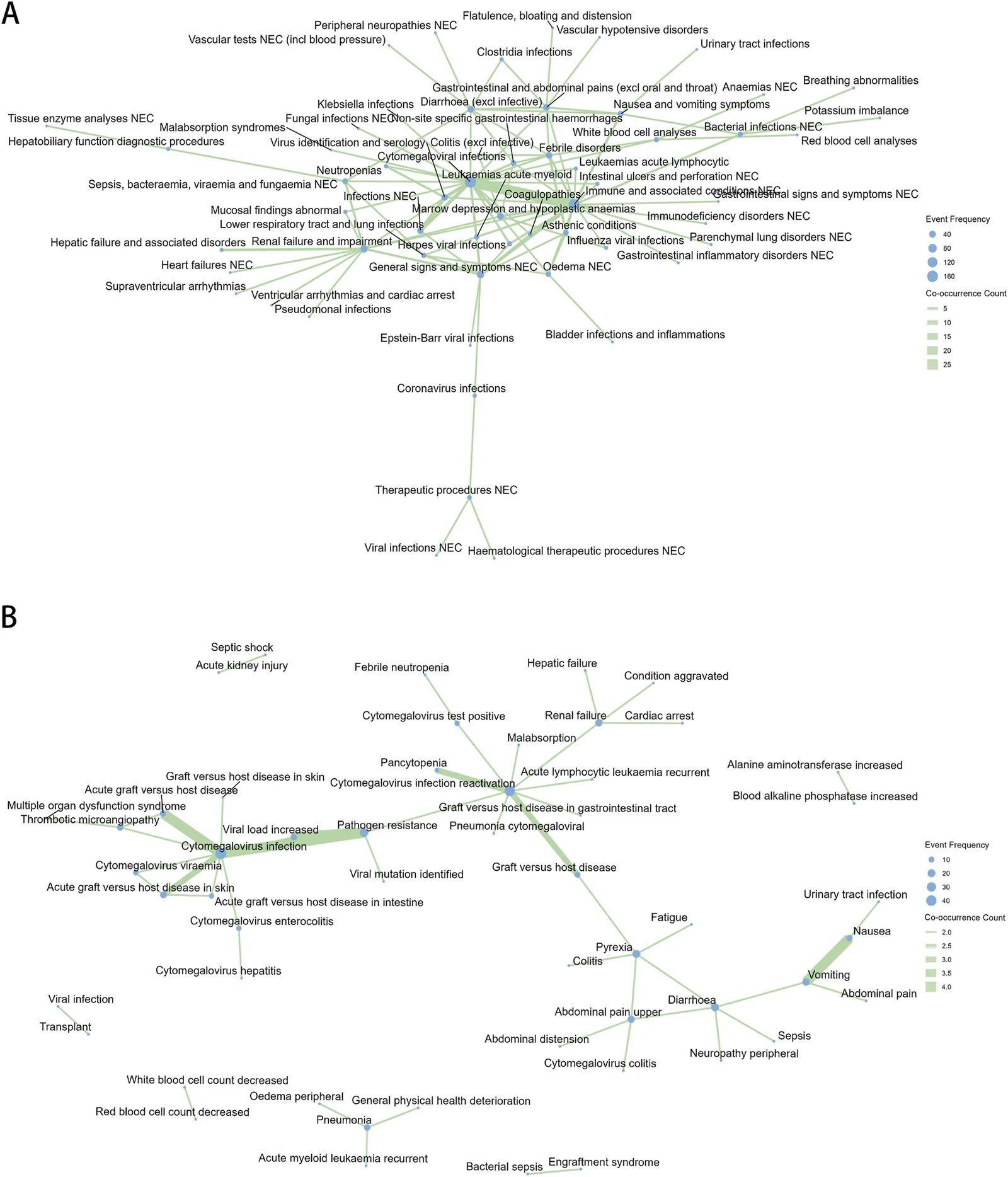
Co-occurrence of ADRs reported with letermovir at HLT **(A)** and PT levels **(B)**. ADRs: adverse drug events; HLT: High-level terms, PT: Preferred terms.

### Disproportionality analysis

Disproportionality analysis included events with ≥3 reports to identify safety signals. When comparing letermovir to all other drugs, 3 significant signals were detected at SOC level (Figures 4A,D; Supplementary Table S4), 24 at HLT level (Figures 4B,E; Supplementary Table S5), and 47 at PT level (Figures 4C,F; Supplementary Table S6). On the other hand, comparisons restricted to drugs used in BMT revealed 1 significant SOC signal (Figures 5A,D; Supplementary Table S7), 4 HLT signals (Figures 5B,E; Supplementary Table S8), and 6 PT signals (Figures 5C,F; Supplementary Table S9). Venn plots illustrated the overlaps of significant signals between these two analyses (Figure 6). At the SOC level, three signals were identified in the overall analysis, whereas only surgical and medical procedures remained with the BMT comparator. At the HLT level, cytomegaloviral infections, virus identification and serology, and hematological therapeutic procedures were shared between analyses. At the PT level, all six BMT-comparator signals overlapped with the overall analysis: dysphagia, CMV infection, CMV infection reactivation, pathogen resistance, engraft failure, and stem cell transplant. The CMV infection, reactivation, and pathogen-resistance signals may reflect reports of breakthrough or recurrent CMV-related events during prophylaxis rather than drug effects, whereas engraft failure and stem cell transplantation may reflect the underlying transplant procedure. In the overall analysis, atrial fibrillation and oedema were consistent with advese events described in the prescribing information. Additional signals included hematological abnormalities (agranulocytosis, pancytopenia, and thrombotic microangiopathy), pericardial effusion, dysphagia, stomatitis, hepatic and renal failure, bacterial and viral infections, including EBV and HHV-6 related events, septic shock and viral mutation. Transplantation associated signals included GVHD, engraft failure, acute myeloid leukemia recurrence and transplantation procedures. These findings only represent disproportionality signals and thus require clinical validation.

**FIGURE 4:**
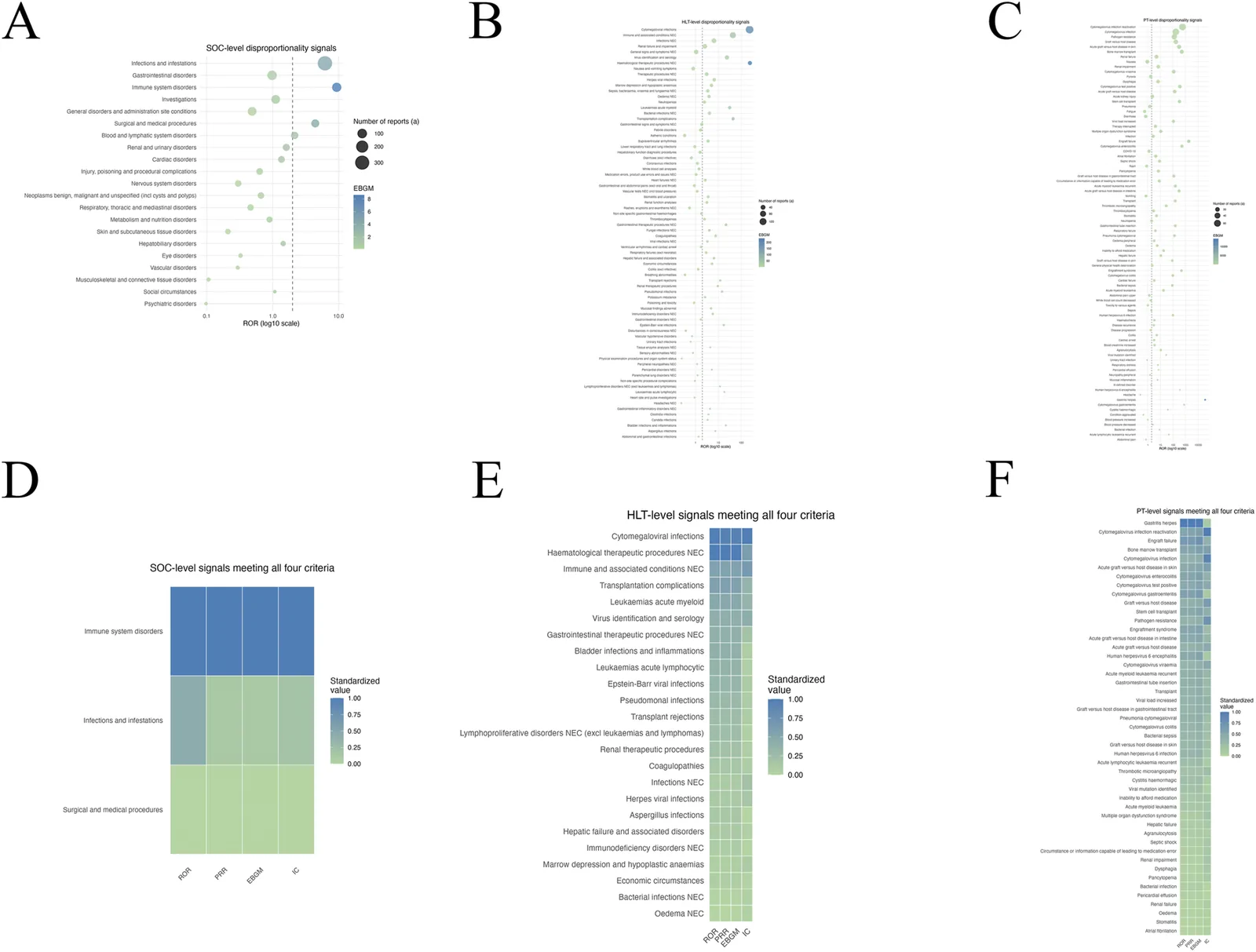
Disproportionality analysis of letermovir compared to all others drugs. **(A–C)** showed reports number, ROR and EBGM for events with ≥3 reports at SOC, HLT and PT level, respectively. Heatmaps **(D–F)** displayed scaled ROR, PRR, IC and EBGM of significant signals at SOC, HLT and PT level, respectively. ROR, Reporting Odds Ratio; EBGM, Empirical Bayes Geometric Mean; ADRs, adverse drug events; SOC, System Organ Class, HLT, High-Level Term; PT, Preferred Term; PRR, Proportional Reporting Ratio; IC, Information Component.

**FIGURE 5:**
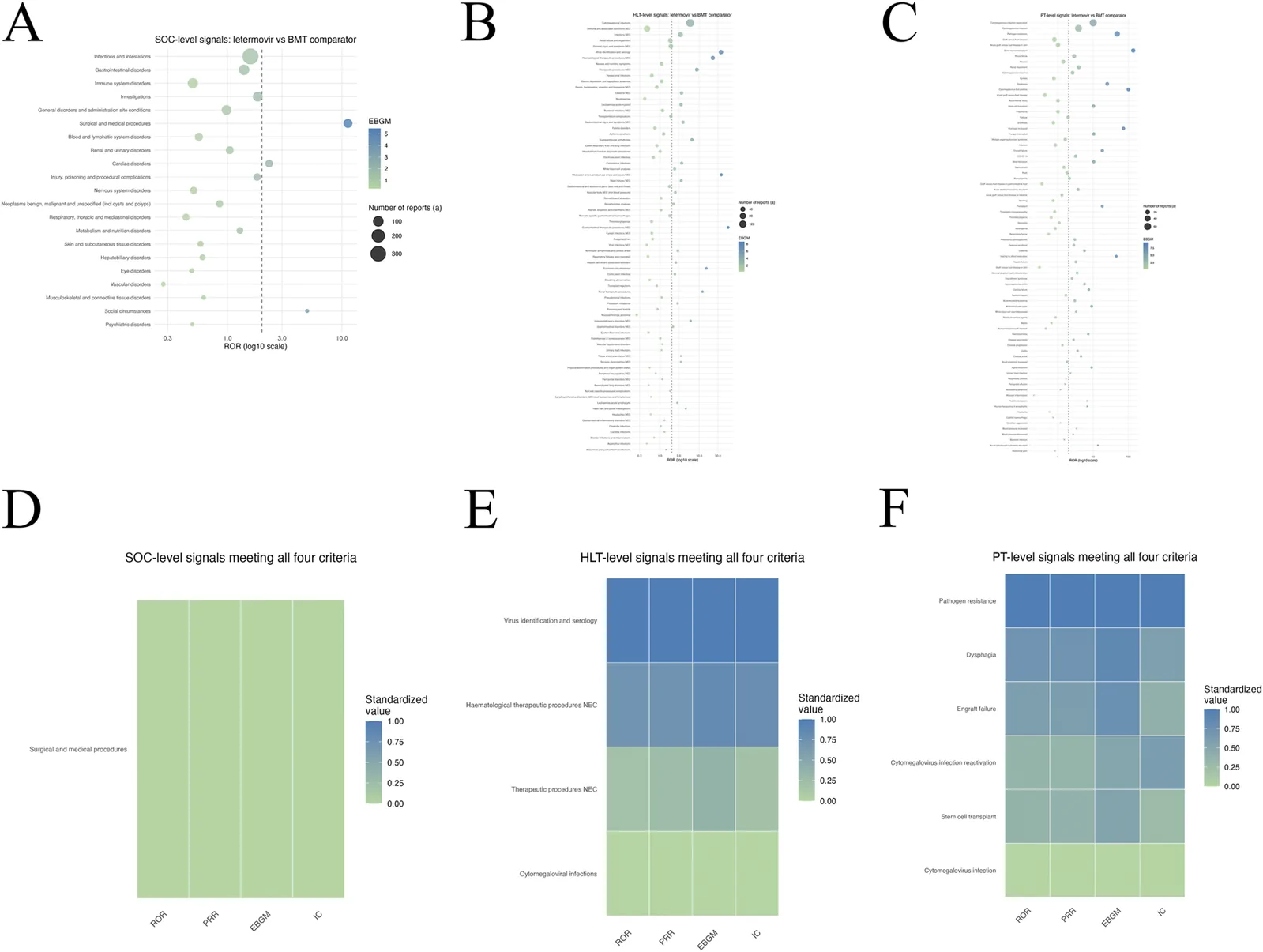
Disproportionality analysis of letermovir compared to others drugs used for bone marrow transplant. **(A–C)** showed reports number, ROR and EBGM for events with ≥3 reports at SOC, HLT and PT level, respectively. Heatmaps **(D–F)** displayed scaled ROR, PRR, IC and EBGM of significant signals at SOC, HLT and PT level, respectively. ROR, Reporting Odds Ratio; EBGM, Empirical Bayes Geometric Mean; ADRs, adverse drug events; SOC, System Organ Class, HLT, High-Level Term; PT, Preferred Term; PRR, Proportional Reporting Ratio; IC, Information Component.

**FIGURE 6:**
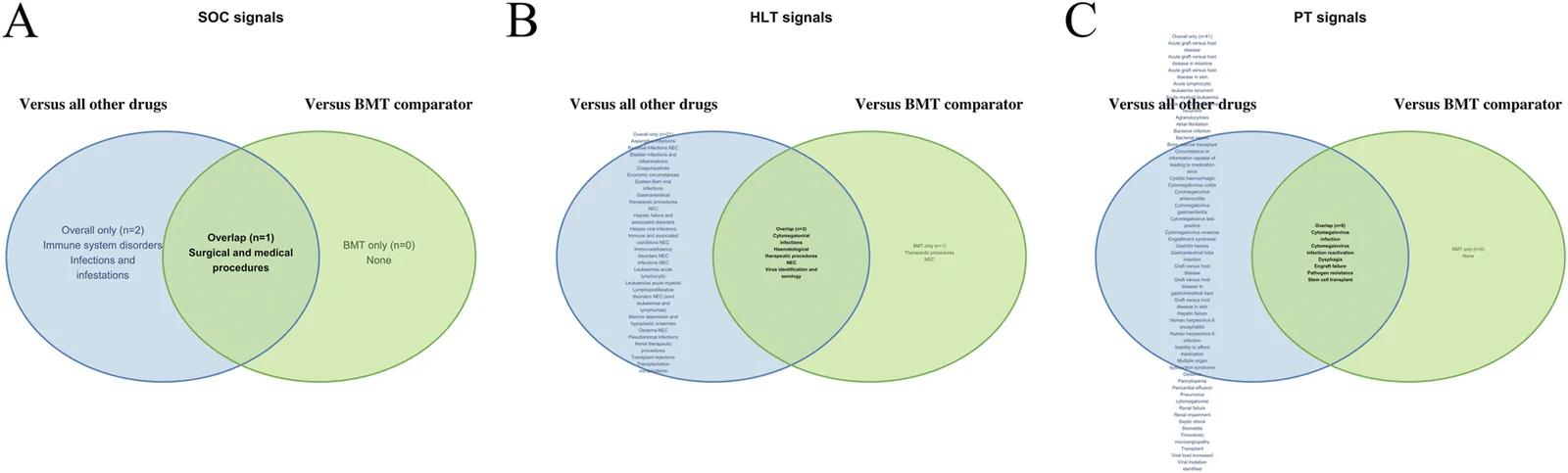
Overlap between significant signals from two disproportionality analyses at System Organ Class **(A)**, High-Level Terms **(B)** and Preferred Terms levels **(C)** in letermovir reports.

### Time-to-onset and report level associations analysis

Only reports with available treatment initiation and event onset dates were included for time-to-onset analysis. The median recorded time to onset for all reported ADRs was 25 (95% CI: 17–28) days (Figure 7A). Tablet reports involved older age, higher weight, a higher proportion of male gender, a greater representation from South America, and lower representation from Europe (Table 4). Reports involving intravenous formulation showed a longer recorded onset time of 33 (95% CI: 26–109) days, compared to 17 (95% CI: 17–25) days for tablet (P = 0.009) (Figure 7B). Among CMV-related reports during letermovir treatment, the median recorded time to onset was 33 (95% CI: 13–109) days (Figure 7C). Tablet-related CMV reports had a higher proportion of male gender and greater representation from Asia, while intravenous-related reports were more frequently submitted from Europe (Table 4). Recorded onset of CMV-related events was later in intravenous than tablet reports (median: 110 (95% CI: 33–124) days *versus* 13 (95% CI: 8–56) days, P = 0.027) (Figure 7D). In exploratory univariable logistic regression analyses (Figure 8), older age was associated with lower odds of CMV-related event reporting (OR = 0.981, 95% CI: 0.968–0.994). Intravenous administration was associated with higher reporting odds than tablet administration (OR = 1.685, 95% CI: 1.034–2.744), while more recent treatment initiation was associated with lower reporting odds (OR = 0.813, 95% CI: 0.686–0.964). Compared with Asia, reports from North America showed lower odds of CMV-related events reporting (OR = 0.108, 95% CI: 0.053–0.221). These findings represent exploratory report-level associations rather than clinical risk estimates.

**FIGURE 7:**
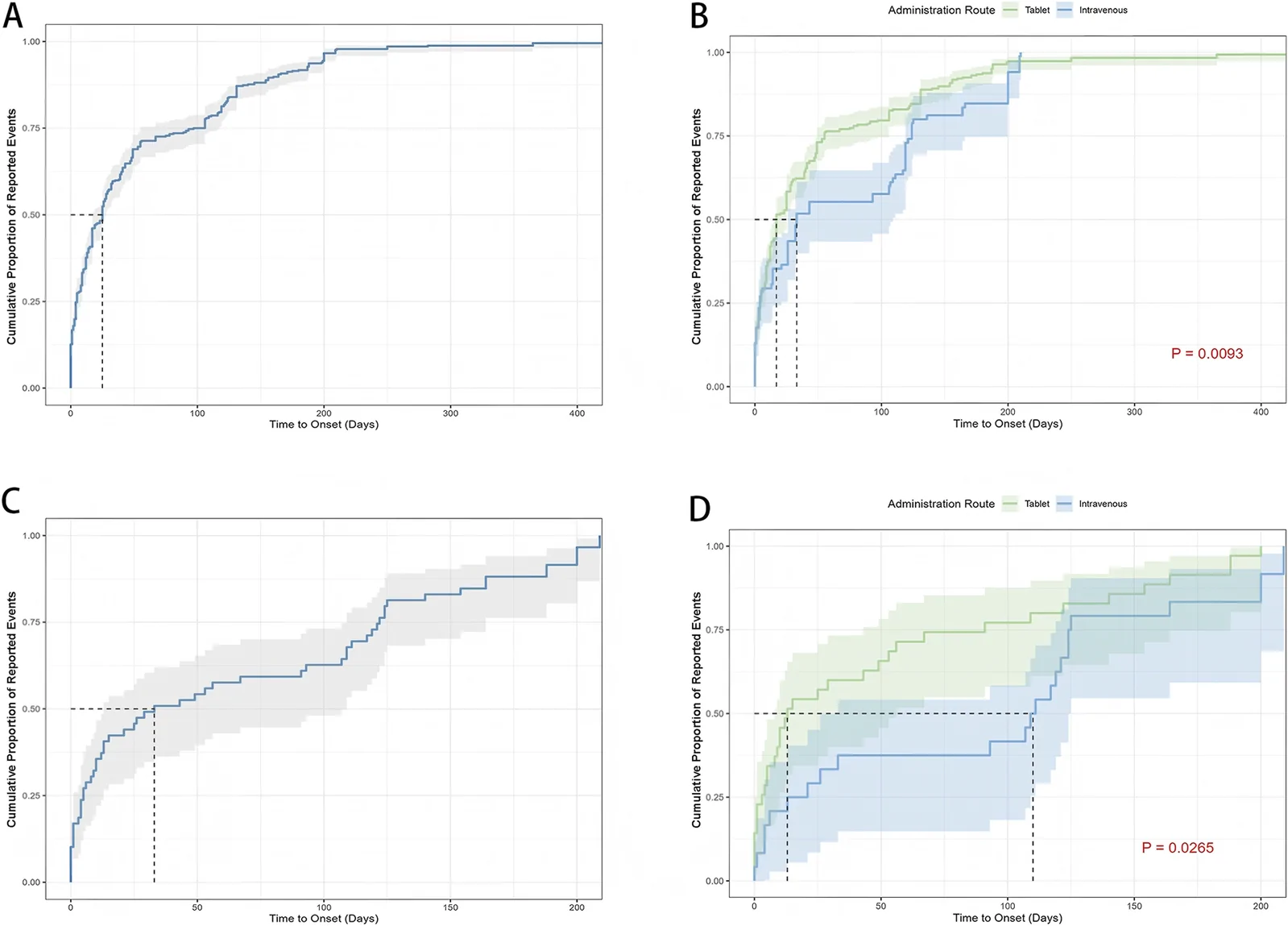
Cumulative distribution of recorded time to onset among letermovir reports. **(A,C)** showed cumulative distribution of all reports **(A)** and CMV-related events **(C)** during letermovir treatment. **(B,D)** illustrated stratification by administration route (tablet vs. intravenous) for all reports **(B)** and CMV-related events **(D)** during letermovir treatment.

**TABLE 4:** Description of reports for two dose forms.

Overall adverse drug reaction				CMV-related adverse drug reaction		
Item	Tablet (n = 465)	Intravenous (n = 121)	P value	Tablet (n = 49)	Intravenous (n = 26)	P value
Age (years), median (range)	55 (7-78)	45 (0.5-72)	<0.001	50 (17-70)	39.5 (2-68)	0.080
Gender			0.038			0.058
Male	219 (47.1%)	45 (37.2%)	0.042	30 (61.2%)	9 (34.6%)	0.028
Female	241 (51.8%)	72 (59.5%)	0.163	18 (36.7%)	16 (61.5%)	0.040
Unknown	4 (0.9%)	5 (4.1%)	0.028	1 (2.0%)	1 (3.8%)	0.644
Weight (kg)	59 (22-118)	51 (10-100)	0.002	54 (34.5-95)	50.25 (10-100)	0.559
Event year						
Median	2020 (2017-2024)	2020 (2018-2024)	0.319	2019 (2018-2024)	2019 (2018-2021)	0.319
2017	15 (3.3%)	0 (0)		13 (27.1%)	3 (11.5%)	
2018	112 (24.7%)	29 (25.0%)		11 (22.9%)	12 (46.2%)	
2019	69 (15.2%)	17 (14.7%)		5 (10.4%)	6 (23.1%)	
2020	69 (15.2%)	35 (30.2%)		7 (14.6%)	5 (19.2%)	
2021	80 (17.6%)	19 (16.4%)		7 (14.6%)	0 (0)	
2022	38 (8.4%)	11 (9.5%)		3 (6.3%)	0 (0)	
2023	63 (13.9%)	3 (3.4%)		2 (4.2%)	0 (0)	
2024	8 (1.8%)	1 (0.9%)				
Occurring country						
North America	89 (19.1%)	17 (14.0%)	0.195	3 (0.1%)	0 (0)	0.198
South America	15 (3.2%)	0 (0)	0.045	1 (2.0%)	0 (0)	0.463
Europe	148 (31.8%)	56 (46.3%)	0.002	12 (24.5%)	19 (73.1%)	<0.001
Asia	213 (45.8%)	48 (39.7%)	0.226	33 (67.3%)	7 (26.9%)	<0.001

**FIGURE 8:**
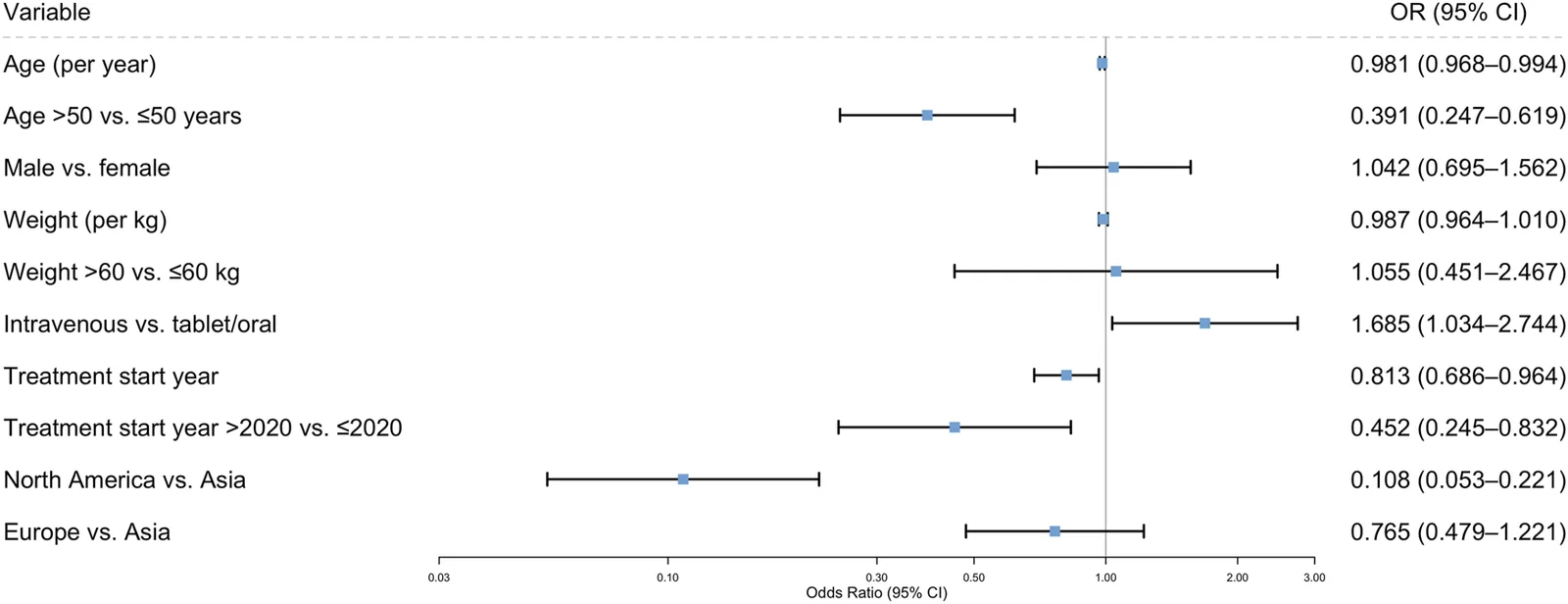
Exploratory univariable logistic regression analysis of factors associated with CMV-related events reported during letermovir treatment.

## Discussion

Letermovir represents a significant advancement in CMV management by providing targeted prophylaxis, shifting the strategy from preemptive therapy to prevention. It effectively suppresses CMV reactivation in transplant recipients, demonstrating superior safety and efficacy. However, ongoing monitoring remains crucial to characterize potential safety signals and breakthrough infections. In this study, we conducted a comprehensive analysis of events reported during letermovir treatment using the FAERS dataset, focusing specifically on CMV-reltaed events and the detection of safety signals *via* disproportionality analysis. Our analysis identified reporting signals not included in the prescribing information and explored report-level factors associated with CMV-related events. These results provide additional insights into the real-world safety profile of letermovir, indicating potential areas for enhanced pharmacovigilance and clinical investigation.

Among the most frequently reported events, infections and infestations were particularly prominent, with cytomegaloviral infections representing the most frequent events at the HLT and PT levels. Additionally, disproportionality analyses using two different comparator groups consistently identified CMV infections as significant signals at both PT and HLT levels. Because letermovir is indicated for CMV prophylaxis, these signals may reflect breakthrough infection, prophylactic failure, or less likely confounding by indication or reporter coding practices. Nevertheless, CMV viraemia and CMV colitis reported during treatment indicate that breakthrough CMV-related events remain clinically relevant. Potential contributing factors include the emergence of CMV resistance mutations (Robin et al., 2020; Muller et al., 2022; Chou, 2015), consistent with disproportionality signals for pathogen resistance and the co-occurrence of CMV infection/reactivation with pathogen resistance in our analysis. Letermovir resistance has been observed in both SOT and HSCT recipients (Perchetti et al., 2023; Aryal et al., 2019; Hofmann et al., 2021). Although resistance was typically manageable with alternative regimens and not a major concern in most cases (Perchetti et al., 2023; Walti et al., 2023), one study suggested potential associations between resistance and factors such as treatment interruption or suboptimal letermovir concentration, underscoring the need for therapeutic drug monitoring and genotyping (Alain et al., 2020). Additional factors for CMV breakthrough infection during letermovir therapy included low-grade CMV replication, acute gastrointestinal GVHD and cumulative steroid exposure (Royston et al., 2021; Zamora et al., 2021b). Notably, the frequent co-occurrence of CMV infections with immune dysregulation conditions, particularly GVHD in our study, is consistent with previous studies (Bueno et al., 2021; Suessmuth et al., 2020; Cantoni et al., 2010). However, the definitive causal relationship remains undetermined: some evidence indicated that GVHD may predispose patients to CMV infection *via* altered immune reconstitution (Suessmuth et al., 2020; Ljungman et al., 2006), while other studies demonstrated that CMV infection may upregulate inflammatory responses associated with GVHD (Palaniyandi et al., 2013; Sen et al., 2020). This complex bidirectional interplay indicates the importance of vigilant monitoring for both CMV breakthrough infection and immune-related complications in patients receiving letermovir.

Disproportionality analyses using all other drugs as comparator group identified several reporting signals that are not included in the letermovir prescribing information. For cardiac complications, while tachycardia and atrial fibrillation are mentioned in prescribing information, our study identified safety signals for pericardial effusion. Both SOT and HSCT recipients have elevated baseline risks of cardiac complications (Wyld et al., 2021; Aghaulor and VanWagner, 2021; Ataş et al., 2020). According to previous studies, cardiac complications following HSCT were associated with older age, higher creatinine, allo-HSCT, cumulative doxorubicin dosage, GVHD severity and baseline cardiovascular comorbidities (Vasbinder et al., 2023; Baker et al., 2020; Moriyama et al., 2022). Similarly, age, kidney function and baseline cardiovascular conditions were also identified as risk factors in SOT recipients (Birdwell and Park, 2021; Munagala and Phancao, 2016). However, prior clinical research reported no severe cardiovascular complications attributable to letermovir, and the drug was considered safe in heart transplant patients (Boignard et al., 2022; Golob et al., 2022). Therefore, these signals may reflect the underlying transplant population, comorbidities, or concomitant treatment rather than direct letermovir toxicity and require clinical validation. Another noteworthy finding was the detection of signals for renal impairment, renal failure and hepatic failure. As increased serum creatinine was reported in allo-HSCT and kidney transplant patients in the product information, and transplant recipients are often at increased risk for renal dysfunction due to calcineurin inhibitors and other nephrotoxic drugs. Thus, further clinical studies are needed to inform whether renal function should be more closely monitored in patients receiving both letermovir and nephrotoxic agents. The detection of hepatic failure also warrants further investigation, as only transient mild transaminitis was reported previously (Kuhn et al., 2023). Letermovir undergoes minimal hepatic metabolism *via* UGT1A1/1A3 enzymes but interacts with multiple metabolic pathways and transporters. It acts as a substrate for CYP3A4, CYP2D6, UGT1A1/1A3, P-glycoprotein, and OATP1B1/3 transporters, while also inhibiting CYP2C8, P-glycoprotein, OATP1B1/3, and moderately inhibiting CYP3A4 (Shigle et al., 2024). Consequently, the renal and hepatic signals from FAERS database may reflect underlying illness, concomitant medications, or drug-drug interactions rather than direct organ toxicity.

AML recurrence was identified as a reporting signal, however, this finding may be subject to substantial bias and should be interpreted with considerable caution. A previous clinical trial similarly observed a numerically higher incidence of AML relapse in letermovir group (6%) compared to placebo group (1%) (Russo et al., 2024), though statistical significance was not reached, and no trend was observed between timing of relapse and treatment duration. Previous studies have reported complex and heterogeneous associations between CMV reactivation, immune reconstitution and leukemia relapse after HSCT (Akahoshi et al., 2023; Turki et al., 2022; Elmaagacli et al., 2011; Semmes et al., 2020; Luo et al., 2022; Millar et al., 2020; Okpoluaefe et al., 2024; Lopez-Vergès et al., 2011). However, analyses using FAERS data cannot establish whether letermovir influences antileukemic immune surveillance, and this signal is more likely to reflect underlying disease, transplantation-related factors, treatment indication, or reporting errors rather than an effect of letermovir. Among other unexpected findings, engraft failure and transplant-related procedures (transplant, stem cell transplant and bone marrow transplant) were detected. Previous studies have implicated CMV infection, even in a latent state, as a risk factor for graft failure (Baldo et al., 2021; Hancock et al., 2020), though our analysis did not observe concurrent CMV infection in these cases. Whether these signals reflect CMV-related complications or incidental occurrences remains unclear due to limited clinical information. Finally, recent studies reported increased EBV reactivation following letermovir prophylaxis in umbilical cord blood transplantation and haploidentical HSCT (Yan et al., 2024; Kong et al., 2024), and our analysis also identified an EBV-related reporting signal at the HLT level. However, our findings do not establish a causal association with letermovir.

Our time-to-onset analysis revealed earlier recorded onset of both overall and CMV-related events in tablet reports than in intravenous reports. Previous studies showed that plasma letermovir concentrations were not lower following intravenous administration compared to oral dosage, and the concentration-time-profile was similar for both formulations (Asari et al., 2022; Prohn et al., 2021). This difference should not be interpreted as a route-specific pharmacological effect because intravenous letermovir is generally used when oral administration is not feasible and may identify patients with greater illness severity or gastrointestinal dysfunction. In exploratory logistic regression, intravenous administration was associated with higher odds of CMV-related event reporting, whereas older age and later treatment initiation were associated with lower reporting odds. Reports from North America also showed lower reporting odds than reports from Asia, while the difference between Europe and Asia was not statistically significant. Although prior research has also demonstrated the racial effects on letermovir exposure (Asari et al., 2022; Prohn et al., 2021), these findings may reflect channeling bias, market differences, regional reporting practices, and other unmeasured factors rather than clinical effects of formulation or geography.

Our study has several limitations inherent to the FAERS database, including potential underreporting, reporting bias, and incomplete clinical data. A key limitation is the inability to establish causality, as disproportionality analysis identifies reporting associations rather than definitive cause-effect relationships. Additionally, the lack of detailed information on concomitant medications, dosing regimens, and underlying patient conditions limits our ability to control for confounding factors and thoroughly evaluate associations. Important transplant-related variables, including transplantation type, donor and recipient CMV serostatus, conditioning regimen, GVHD prophylaxis, corticosteroid exposure and other relevant clinical variables, were unavailable. Also, confounding by indication remains unavoidable. Furthermore, when comparing letermovir to all drugs, there is significant patient variability, although additional clinical context was provided by comparing with BMT drugs specifically. However, the BMT restricted comparator may not capture all HSCT-related reports and should not be regarded as a fully matched clinical cohort. Finally, the time-to-onset analyses were restricted to reports with complete dates, and the univariable regression analyses may involve unmeasured confounding due to limited clinical data provided by FAERS. Despite these limitations, FAERS database remains a valuable tool for post-marketing surveillance. It allows for large-scale data processing and signal detection and may identify uncommon reporting events that may not be noticed in clinical trials. This can provide valuable insights for future clinical research and safety signal investigation.

In conclusion, our analysis identified safety signals reported with letermovir, including both known ADRs and previously unrecognized signals. Exploratory analyses also identified report-level differences according to age, administration route, calendar year, and geographic region, however, these should not be interpreted as clinical risk factors without further validation. These findings underscore the need for ongoing pharmacovigilance and further research to clarify the clinical relevance of these safety signals and optimize the safe use of letermovir in clinical practice.

## Data Availability

The raw data supporting the conclusions of this article will be made available by the authors, without undue reservation.
