# Supplementary material for: Safety signals and CMV-related events reported during letermovir therapy: a FAERS database study
Source: Front Pharmacol. 2026 Sep 29;17:1935778. doi: 10.3389/fphar.2026.1935778 (PMC13657975; doi:10.3389/fphar.2026.1935778)
Supplement: Supplementary file 1 [file Table1.docx]

| Supplementary Table 1. CMV infections in letermovir ADRs reports | |
| --- | --- |
| Disease Name | Count |
| Cytomegalovirus colitis | 5 |
| Cytomegalovirus enteritis | 1 |
| Cytomegalovirus enterocolitis | 8 |
| Cytomegalovirus gastritis | 2 |
| Cytomegalovirus gastroenteritis | 3 |
| Cytomegalovirus hepatitis | 2 |
| Cytomegalovirus infection | 57 |
| Cytomegalovirus infection reactivation | 62 |
| Cytomegalovirus syndrome | 1 |
| Cytomegalovirus test positive | 1 |
| Cytomegalovirus viraemia | 12 |
| Pneumonia cytomegaloviral | 5 |
| ADRs: Adverse drug reactions. |  |
