# Supplementary material for: Safety signals and CMV-related events reported during letermovir therapy: a FAERS database study
Source: Front Pharmacol. 2026 Sep 29;17:1935778. doi: 10.3389/fphar.2026.1935778 (PMC13657975; doi:10.3389/fphar.2026.1935778)
Supplement: Supplementary file 2 [file Table2.docx]

| Supplementary Table 2. Co-occurrence of HLT | | |
| --- | --- | --- |
| Event 1 | Event 2 | Count (n > 1) |
| Immune and associated conditions NEC | Cytomegaloviral infections | 25 |
| Infections NEC | Cytomegaloviral infections | 7 |
| Infections NEC | Virus identification and serology | 7 |
| Virus identification and serology | Cytomegaloviral infections | 6 |
| Marrow depression and hypoplastic anaemias | Cytomegaloviral infections | 5 |
| General signs and symptoms NEC | Cytomegaloviral infections | 4 |
| Immune and associated conditions NEC | General signs and symptoms NEC | 4 |
| Immune and associated conditions NEC | Marrow depression and hypoplastic anaemias | 4 |
| Nausea and vomiting symptoms | Immune and associated conditions NEC | 4 |
| Febrile disorders | Asthenic conditions | 4 |
| Renal failure and impairment | Hepatic failure and associated disorders | 4 |
| Herpes viral infections | Cytomegaloviral infections | 3 |
| Non-site specific gastrointestinal haemorrhages | Cytomegaloviral infections | 3 |
| Immune and associated conditions NEC | Herpes viral infections | 3 |
| Renal failure and impairment | General signs and symptoms NEC | 3 |
| Lower respiratory tract and lung infections | General signs and symptoms NEC | 3 |
| Neutropenias | Virus identification and serology | 3 |
| Febrile disorders | Immune and associated conditions NEC | 3 |
| Bacterial infections NEC | Immune and associated conditions NEC | 3 |
| Leukaemias acute myeloid | Immune and associated conditions NEC | 3 |
| Renal failure and impairment | Sepsis, bacteraemia, viraemia and fungaemia NEC | 3 |
| Gastrointestinal and abdominal pains (excl oral and throat) | Nausea and vomiting symptoms | 3 |
| Gastrointestinal and abdominal pains (excl oral and throat) | Diarrhoea (excl infective) | 3 |
| White blood cell analyses | Cytomegaloviral infections | 2 |
| Febrile disorders | Cytomegaloviral infections | 2 |
| Sepsis, bacteraemia, viraemia and fungaemia NEC | Cytomegaloviral infections | 2 |
| Leukaemias acute lymphocytic | Cytomegaloviral infections | 2 |
| Leukaemias acute myeloid | Cytomegaloviral infections | 2 |
| Neutropenias | Cytomegaloviral infections | 2 |
| Renal failure and impairment | Cytomegaloviral infections | 2 |
| Fungal infections NEC | Cytomegaloviral infections | 2 |
| Diarrhoea (excl infective) | Cytomegaloviral infections | 2 |
| Coagulopathies | Cytomegaloviral infections | 2 |
| Mucosal findings abnormal | Cytomegaloviral infections | 2 |
| Gastrointestinal and abdominal pains (excl oral and throat) | Cytomegaloviral infections | 2 |
| Malabsorption syndromes | Cytomegaloviral infections | 2 |
| Klebsiella infections | Cytomegaloviral infections | 2 |
| Intestinal ulcers and perforation NEC | Cytomegaloviral infections | 2 |
| General signs and symptoms NEC | Herpes viral infections | 2 |
| Therapeutic procedures NEC | Haematological therapeutic procedures NEC | 2 |
| Renal failure and impairment | Supraventricular arrhythmias | 2 |
| Immune and associated conditions NEC | Immunodeficiency disorders NEC | 2 |
| Marrow depression and hypoplastic anaemias | Oedema NEC | 2 |
| Immune and associated conditions NEC | Oedema NEC | 2 |
| Asthenic conditions | Oedema NEC | 2 |
| Lower respiratory tract and lung infections | Oedema NEC | 2 |
| Bladder infections and inflammations | Oedema NEC | 2 |
| Asthenic conditions | General signs and symptoms NEC | 2 |
| Infections NEC | General signs and symptoms NEC | 2 |
| Leukaemias acute myeloid | General signs and symptoms NEC | 2 |
| Coagulopathies | General signs and symptoms NEC | 2 |
| Coronavirus infections | General signs and symptoms NEC | 2 |
| Epstein-Barr viral infections | General signs and symptoms NEC | 2 |
| Immune and associated conditions NEC | Virus identification and serology | 2 |
| Colitis (excl infective) | Marrow depression and hypoplastic anaemias | 2 |
| Influenza viral infections | Marrow depression and hypoplastic anaemias | 2 |
| Renal failure and impairment | Marrow depression and hypoplastic anaemias | 2 |
| Non-site specific gastrointestinal haemorrhages | Marrow depression and hypoplastic anaemias | 2 |
| Febrile disorders | Colitis (excl infective) | 2 |
| Influenza viral infections | Immune and associated conditions NEC | 2 |
| Asthenic conditions | Immune and associated conditions NEC | 2 |
| Gastrointestinal signs and symptoms NEC | Immune and associated conditions NEC | 2 |
| Leukaemias acute lymphocytic | Immune and associated conditions NEC | 2 |
| Renal failure and impairment | Immune and associated conditions NEC | 2 |
| Coagulopathies | Immune and associated conditions NEC | 2 |
| Gastrointestinal inflammatory disorders NEC | Immune and associated conditions NEC | 2 |
| Parenchymal lung disorders NEC | Immune and associated conditions NEC | 2 |
| Gastrointestinal and abdominal pains (excl oral and throat) | Immune and associated conditions NEC | 2 |
| Non-site specific gastrointestinal haemorrhages | Immune and associated conditions NEC | 2 |
| Intestinal ulcers and perforation NEC | Immune and associated conditions NEC | 2 |
| Febrile disorders | White blood cell analyses | 2 |
| Red blood cell analyses | White blood cell analyses | 2 |
| Anaemias NEC | White blood cell analyses | 2 |
| Diarrhoea (excl infective) | Febrile disorders | 2 |
| Gastrointestinal and abdominal pains (excl oral and throat) | Febrile disorders | 2 |
| Non-site specific gastrointestinal haemorrhages | Febrile disorders | 2 |
| Hepatobiliary function diagnostic procedures | Sepsis, bacteraemia, viraemia and fungaemia NEC | 2 |
| Diarrhoea (excl infective) | Sepsis, bacteraemia, viraemia and fungaemia NEC | 2 |
| Lower respiratory tract and lung infections | Sepsis, bacteraemia, viraemia and fungaemia NEC | 2 |
| Tissue enzyme analyses NEC | Hepatobiliary function diagnostic procedures | 2 |
| Bacterial infections NEC | Nausea and vomiting symptoms | 2 |
| Diarrhoea (excl infective) | Nausea and vomiting symptoms | 2 |
| Urinary tract infections | Nausea and vomiting symptoms | 2 |
| Potassium imbalance | Bacterial infections NEC | 2 |
| Breathing abnormalities | Bacterial infections NEC | 2 |
| Diarrhoea (excl infective) | Vascular tests NEC (incl blood pressure) | 2 |
| Lower respiratory tract and lung infections | Leukaemias acute myeloid | 2 |
| Pseudomonal infections | Renal failure and impairment | 2 |
| Heart failures NEC | Renal failure and impairment | 2 |
| Mucosal findings abnormal | Renal failure and impairment | 2 |
| Ventricular arrhythmias and cardiac arrest | Renal failure and impairment | 2 |
| Peripheral neuropathies NEC | Diarrhoea (excl infective) | 2 |
| Non-site specific gastrointestinal haemorrhages | Diarrhoea (excl infective) | 2 |
| Clostridia infections | Diarrhoea (excl infective) | 2 |
| Viral infections NEC | Therapeutic procedures NEC | 2 |
| Coronavirus infections | Therapeutic procedures NEC | 2 |
| Flatulence, bloating and distension | Gastrointestinal and abdominal pains (excl oral and throat) | 2 |
| Vascular hypotensive disorders | Gastrointestinal and abdominal pains (excl oral and throat) | 2 |
| Clostridia infections | Gastrointestinal and abdominal pains (excl oral and throat) | 2 |
