# Supplementary material for: Safety signals and CMV-related events reported during letermovir therapy: a FAERS database study
Source: Front Pharmacol. 2026 Sep 29;17:1935778. doi: 10.3389/fphar.2026.1935778 (PMC13657975; doi:10.3389/fphar.2026.1935778)
Supplement: Supplementary file 3 [file Table3.docx]

| Supplementary Table 3. Co-occurrence of PT | | |
| --- | --- | --- |
| Event 1 | Event 2 | Count (n > 1) |
| Pathogen resistance | Cytomegalovirus infection | 4 |
| Acute graft versus host disease | Cytomegalovirus infection | 4 |
| Viral load increased | Pathogen resistance | 4 |
| Vomiting | Nausea | 4 |
| Acute graft versus host disease in skin | Cytomegalovirus infection | 3 |
| Cytomegalovirus infection reactivation | Graft versus host disease | 3 |
| Pancytopenia | Cytomegalovirus infection reactivation | 3 |
| Cytomegalovirus infection reactivation | Pneumonia cytomegaloviral | 2 |
| Acute graft versus host disease in intestine | Cytomegalovirus infection | 2 |
| Thrombotic microangiopathy | Cytomegalovirus infection | 2 |
| Viral load increased | Cytomegalovirus infection | 2 |
| Cytomegalovirus enterocolitis | Cytomegalovirus infection | 2 |
| Cytomegalovirus viraemia | Cytomegalovirus infection | 2 |
| Graft versus host disease in skin | Cytomegalovirus infection | 2 |
| Febrile neutropenia | Cytomegalovirus test positive | 2 |
| Cytomegalovirus infection reactivation | Cytomegalovirus test positive | 2 |
| Pyrexia | Colitis | 2 |
| Pyrexia | Graft versus host disease | 2 |
| Pneumonia | Oedema peripheral | 2 |
| Diarrhoea | Pyrexia | 2 |
| Fatigue | Pyrexia | 2 |
| Abdominal pain upper | Pyrexia | 2 |
| Cytomegalovirus infection reactivation | Pathogen resistance | 2 |
| Viral mutation identified | Pathogen resistance | 2 |
| Cytomegalovirus infection reactivation | Graft versus host disease in gastrointestinal tract | 2 |
| Renal failure | Hepatic failure | 2 |
| Diarrhoea | Sepsis | 2 |
| Acute graft versus host disease in skin | Acute graft versus host disease in intestine | 2 |
| Cytomegalovirus infection reactivation | Acute lymphocytic leukaemia recurrent | 2 |
| Urinary tract infection | Nausea | 2 |
| Engraftment syndrome | Bacterial sepsis | 2 |
| Pneumonia | General physical health deterioration | 2 |
| Pneumonia | Acute myeloid leukaemia recurrent | 2 |
| Neuropathy peripheral | Diarrhoea | 2 |
| Vomiting | Diarrhoea | 2 |
| Abdominal pain upper | Diarrhoea | 2 |
| Acute graft versus host disease | Thrombotic microangiopathy | 2 |
| Multiple organ dysfunction syndrome | Thrombotic microangiopathy | 2 |
| Septic shock | Acute kidney injury | 2 |
| Cytomegalovirus viraemia | Acute graft versus host disease in skin | 2 |
| Renal failure | Cytomegalovirus infection reactivation | 2 |
| Malabsorption | Cytomegalovirus infection reactivation | 2 |
| Cytomegalovirus hepatitis | Cytomegalovirus enterocolitis | 2 |
| Cardiac arrest | Renal failure | 2 |
| Condition aggravated | Renal failure | 2 |
| Blood alkaline phosphatase increased | Alanine aminotransferase increased | 2 |
| Viral infection | Transplant | 2 |
| Vomiting | Abdominal pain | 2 |
| Abdominal pain upper | Cytomegalovirus colitis | 2 |
| Abdominal pain upper | Abdominal distension | 2 |
| White blood cell count decreased | Red blood cell count decreased | 2 |
